# Health-related quality of life, temperament, and eating behavior among formula-fed infants in the Philippines: a pilot study

**DOI:** 10.1186/s12955-018-0944-5

**Published:** 2018-06-08

**Authors:** Sheri Volger, Elvira M. Estorninos, Maria R. Capeding, Jowena Lebumfacil, Diane Rigassio Radler, J. Scott Parrott, Pamela Rothpletz-Puglia

**Affiliations:** 1 Rutgers University School of Health Professions, Department of Clinical and Preventive Nutrition Sciences, 65 Bergen Street, Newark, NJ 07107 USA; 2 Nestlé Nutrition Research, King of Prussia, PA USA; 3 Asian Hospital & Medical Center, Medical Office Building, 2205 Civic Drive, Filinvest Corporate City Alabang, 1708 Muntinlupa City, Philippines; 4 Wyeth Philippines Inc, 8 Rockwell, Hidalgo Drive, Rockwell Center, Makati City, Philippines

**Keywords:** Health-related quality of life, HRQoL, Infant quality of life, The infant toddler quality of life questionnaire, Infant characteristics questionnaire, Baby eating behaviour questionnaire, Infant eating behavior, Infant temperament

## Abstract

**Background:**

The rising prevalence of childhood obesity in Asia has led to interest in potential risk factors such as infant health-related quality of life (HRQoL), temperament and eating behaviors. This pilot study evaluated the utility of administering parent-reported outcome measures (PROMs) to explore these factors in Filipino infants and examined the relationships between these factors and infant sex, formula intake and weight, over time.

**Methods:**

Forty healthy, 4-week-old, formula-fed infants (*n* = 20 males) were enrolled in this 6-week, prospective, uncontrolled study during which infants were exclusively fed a standard term infant formula enriched with alpha-lactalbumin. On Day-1 and 42, anthropometrics were measured and mothers completed a 97-item measure of HRQoL [Infant Toddler Quality of Life Questionnaire (ITQOL)] covering 6 infant-focused and 3 parent-focused concepts and a 24-item measure of infant temperament [Infant Characteristics Questionnaire (ICQ)]. At Day-42, mothers also completed an 18-item measure of infant appetite [Baby Eating Behaviour Questionnaire (BEBQ)]. A 3-day formula intake diary was completed before Day-42. Nonparametric statistics were used to evaluate correlations among outcomes and compare outcomes by visit and sex.

**Results:**

Thirty-nine infants completed the study; similar results were observed in males and females. Completion of PROMs was 100% with no missing responses, but Cronbach’s α was low for many concept scales scores. ITQOL scores [range 0 (worst)-100 (best)] were generally high (median ≥ 80) except for Day-1 and Day-42 Temperament and Mood and Day-1 General Health Perceptions scores. ITQOL but not ICQ temperament scores improved significantly between Day-1 and Day-42 (*P* < 0.01). Mean ± standard deviation BEBQ scores (range 1–5) were high for Enjoyment of Food (4.59 ± 0.60) and Food Responsiveness (3.53 ± 0.81), and low for Satiety Responsiveness (2.50 ± 0.73) and Slowness in Eating (1.71 ± 0.60). Better HRQoL scores were significantly (*P* < 0.05) associated with high General Appetite scores (3 ITQOL concepts, *r* = 0.32 to 0.54), greater Enjoyment of Food (4 ITQOL concepts, *r* = 0.35 to 0.42) and low levels of Slowness in Eating (7 ITQOL concepts, *r* = − 0.32 to − 0.47).

**Conclusion:**

Findings demonstrated the utility of the ITQOL, ICQ and BEBQ for measuring HRQoL, temperament and eating behavior, and the need for further adaptations for use in Filipino infants.

**Trial registration:**

ClinicalTrials.gov identifier NCT02431377; Registered May 1, 2015.

## Background

More than 41 million children under age 5 years worldwide are overweight [defined as weight-for-height greater than 2 standard deviations above the World Health Organization’s (WHO) median growth standard] [1]. In the Philippines, the prevalence of overweight children is increasing [2]. In 2013, the overall prevalence of overweight in Filipino children 0–5 years of age was 5%, with the prevalence varying from 2.8 to 10.5% depending on place of residence and economic status [2]. These rates are concerning as childhood overweight and obesity are associated with both later obesity and an increased disease burden in adulthood [3–6]. Thus, there is growing public interest in halting the rise in childhood obesity [7] and a need for a comprehensive approach to identify culturally appropriate, early childhood interventions to lower the prevalence of childhood overweight and obesity and the subsequent risk of later obesity.

Childhood obesity has been attributed to a series of complex interactions between genetics and the environment [8, 9], Scientific evidence suggests that metabolic programming for an increased risk of obesity may begin prenatally but continues through the postnatal feeding period [9, 10]. However, during the period of time when infants are exclusively milk-fed, parent-child interactions and feeding behaviors also may influence infant growth patterns [11, 12]. Furthermore, cultural beliefs [13–15], infant temperament [16–20], infant behavior (i.e., crying, fussing, distress, sleeping) [21, 22] and the child’s home food environment [23] are potential modifiers of parent-child interactions and feeding practices [14, 24]. For example, in some Asian cultures a large baby may be perceived as a healthy infant and, in China, it is not uncommon for parents of healthy infants and toddlers to identify normal weight children as being underweight [15, 25]. Thus, a culturally biased perception of normal infant growth may result in overfeeding [14, 26]. Local beliefs and traditions may also modify parental response to signals of hunger and satiety influencing feeding practices [27]. An infant perceived as having a difficult temperament may be fed longer or more frequently [15, 28] and parental use of food to soothe infants has been shown to be significantly, positively associated with weight gain [11, 18–20].

Numerous studies have shown that rapid early weight gain in infancy is a risk factor for childhood obesity [29–33]. Few studies, however, have examined the relationship between potential confounders or modifiers of parent-child interactions and feeding practices and the risk of rapid weight gain during infancy and later childhood obesity. Moreover, even less is known about the impact of infant feeding practices and weight gain velocity on the health-related quality of life (HRQoL) of an infant [34–36]. A major obstacle to examining these relationships is the limited availability of culturally adapted, validated, parent-reported outcome measures (PROMs) of relevant endpoints. Furthermore, there is a lack of normative or reference data to facilitate the clinical interpretation of study results obtained using such measurement tools. Finally, the majority of assessment tools have been developed in Western populations, limiting their generalizability to other populations such as those in Asia.

The primary aim of the present study was to assess the utility of PROMs of infant HRQoL, temperament and eating behavior in a Filipino population. The secondary aims were to describe infant HRQoL, temperament and eating behaviors by sex and over time, and to examine the relationships between these factors and infant formula intake and weight. Based on previous literature [17, 37–41] we hypothesized that mothers’ assessments of infant HRQoL would be positively associated with the infant’s enjoyment of food and general appetite, and negatively associated with slowness in eating. We also hypothesized that difficult infant temperament would be associated with eating behaviors reflecting greater food responsiveness and lower food avoidance.

## Methods

This study utilizes data collected during a 6-week, prospective, uncontrolled trial (ClinicalTrials.gov identifier NCT02431377), evaluating GI tolerance, as well as HRQoL, temperament, and eating behaviors in Filipino infants. This paper reports the results for the secondary outcomes, HRQoL, temperament and appetite, while the primary gastrointestinal (GI) outcome is reported in a separate paper.

The study was conducted at the Asian Hospital and Medical Center, Muntinlupa City, Philippines from April 2015 to August 2015. A sample of consecutive male (*n* = 20) and female (n = 20) infants whose parents expressed interest in participating in the study and who met the inclusion and exclusion criteria were recruited at community well-baby clinics. Eligible participants were otherwise healthy, full-term (37 to 42 weeks gestation), singleton infants aged 28 days (± 7 days) with weight-for-age ≥ 5th and ≤ 95th percentile according to World Health Organization Child Growth Standards [42], whose parents had previously made the decision to exclusively formula-feed their infants and mothers who were willing and able to complete PROMs. Infants with any of the following criteria or health conditions were excluded: receiving supplemental breast milk; history of siblings with cow’s milk protein intolerance / allergy; requiring specialized infant feedings; major congenital malformations, systemic or congenital infections; significant cardiac, respiratory, endocrinologic, hematologic, gastrointestinal, or other systemic diseases; participation in any other clinical trial; infant’s receiving any prescription or over-the-counter medication, herbals, pre- or probiotics to treat a known or suspected gastrointestinal condition. Due to the study’s small sample size, only one infant per family was allowed to participate, to minimize selection bias. The study was conducted in accordance with the Declaration of Helsinki and Good Clinical Practices and was approved by the Institutional Review Board and the Philippines Food and Drug Administration. All parent(s) or legally acceptable representative(s) (henceforth “parents” or “mothers”) provided informed consent to participate in trial-related procedures in accordance with applicable regulatory requirements. The informed consent process was fully documented.

Infants were enrolled (Study Day 1) as part of a clinical study examining GI tolerance of a routine use, standard term infant formula enriched with alpha-lactalbumin, and provided infant formula for a period of 42 days. No additional compensation was provided. Mother and infant pairs returned to the study site at Days 14 and 42. A standardized study protocol for collecting outcome data was followed at each study visit.

### Outcome measures

Anthropometry was measured by trained staff members in duplicate at enrollment (Day 1), Day 14 and the final study visit (Day 42). Infant weight was measured without a diaper, on a calibrated electronic infant scale (Seca 334, Hamburg, Germany) to the nearest gram. Recumbent length was measured with a Seca measuring rod to the nearest 0.1 cm. Body mass index (BMI, kg/m^2^) was calculated using body weight and length measurements. Head circumference (occipital frontal circumference) was measured with a pediatric head circumference measuring tape (Seca 212, Hamburg, Germany) to the nearest 0.1 cm.

On Day 1, self-administered, paper-and-pencil versions of the questionnaires were completed by mothers to obtain socio-demographic information and to assess HRQoL and infant temperament. Prior to the Day 42 visit, mothers completed a consecutive three-day feeding diary, designed to prospectively record formula intake. On Day 42, mothers again completed questionnaires to assess HRQoL and infant temperament and also completed a questionnaire to assess infant eating behaviors. Data on infant morbidity and adverse events (AEs) were collected in a standardized fashion through interviews by trained research staff at all visits.

### Infant toddler quality of life questionnaire™ (ITQOL)

The Infant Toddler Quality of Life Questionnaire™ (ITQOL) [43] was linguistically translated according to rigorous international guidelines [44–46] from English into Tagalog and used to assess infant HRQoL on Days 1 and 42. The self-administered questionnaire designed for use in children ages 2 months to 5 years, was administered in our slightly younger infant population, with the developer’s approval. The ITQOL includes 97 items that assess both infant- and parent-focused concepts (only 68 items apply to infants who are less than 1 year of age) and contains 6 scales with a total of 53 items that cover infant-focused concepts, including Overall Health, Physical Abilities, Growth and Development, Bodily Pain/Discomfort, Temperament and Moods, and General Health Perceptions. Additionally, there are 3 scales with a total of 15 items that pertain to parent-focused concepts: Parental Impact-Emotional, Parental Impact-Time and Family Cohesion. The instrument was scored according to the developer’s guidelines [47]. Raw concept scores were transformed to values between 0 and 100, where 0 indicates the worst quality of life score and 100 indicates the highest or best score.

### Infant characteristics questionnaire (ICQ)

Difficult infant temperament was measured on Days 1 and 42 using the Infant Characteristics Questionnaire (ICQ) [48]. The ICQ is a self-reported, 24-item questionnaire validated in a sample of 332 infants 4 to 6 months of age residing in Mid-Western United States (US) that measures parental perceptions of infant temperament. Composite scores for each of the four factor scales (Fussy-Difficult, Unadaptable, Dull, and Unpredictable) were calculated by summing the individual items, which are scored on a 7-point response scale with the lowest ICQ score (1) describing an optimal temperament trait and the highest score (7) indicating a difficult temperament. With permission and advice from the developer [48], the questionnaire was translated into Tagalog and back translated into English to ensure the accuracy and cultural sensitivity of the translation. The forward and back translations were reviewed by experienced medical personnel in the Philippines and members of the research team, and items from the Tagalog translation were revised accordingly.

### Baby eating behaviour questionnaire (BEBQ)

Eating behavior was assessed at the end of the study (Day 42) when the infants were approximately 70 days of age using the linguistically translated [44–46] Tagalog version of the Baby Eating Behaviour Questionnaire (BEBQ) [37, 38]. The BEBQ is a parent-administered 18-item measure of appetite designed for use during the period of time when infants are exclusively human-milk or formula-fed. The questionnaire consists of a single-item measure of general appetite and 4 multi-item appetite trait scales measuring: Enjoyment of Food, Food Responsiveness, Slowness in Eating, and Satiety Responsiveness. The response scale ranges from 1 to 5 (never, rarely, sometimes, often, or always). The instrument was scored according to the developer’s scoring instructions [49]. Scores for the individual appetite traits were calculated by summing the individual item scores and dividing the total by the number of items in the appetite trait. Higher scores indicate that the mother perceives a stronger expression of the specific appetite trait (i.e., greater Enjoyment of Food, higher Food Responsiveness, Slower Eating, higher Satiety Responsiveness and overall greater General Appetite) [50].

#### Sample size determination

The sample size was based on the study’s GI tolerance outcome, the Infant Gastrointestinal Symptom Questionnaire (IGSQ) score, which is reported in a separate publication. Briefly, it was determined that a sample size of 30 subjects was needed to provide at least 90% power to show non-inferiority of IGSQ scores compared to a pre-determined, non-inferiority criterion based on mean IGSQ scores from several previous studies [51, 52] conducted in similar infant populations. Since the focus of the present report is to explore the utility of the PROMs of infant HRQol, temperament, and appetite, a separate sample size calculation for these outcomes was not done. However, in accordance with sample size recommendations for pilot studies [53] and based on a desired expected effect size of 0.5 SD in a future, larger study, our pilot study was appropriately sized (*n* > 15 per arm).

#### Statistical analysis

Data were analyzed using SPSS-21 (version 21; SPSS Inc., Chicago Illinois, USA). Baseline characteristics, including infant anthropometric measurements and infant and parent demographic characteristics, were summarized using descriptive statistics. Continuous variables were reported as mean and standard deviation (SD) or median and interquartile ranges; categorical variables were reported as absolute numbers and percentages (%). Descriptive statistics were computed by visit and sex. The distribution of the data were examined with boxplots, Q_Q plots, and non-normality was confirmed using the Shapiro-Wilks test. Group differences in PROM scores were tested using Mann-Whitney U test. Within-group differences were tested using the Wilcoxon Sign Rank test. To evaluate the internal consistency of instrument scales, Cronbach’s alpha was calculated for each instrument scale with more than one item; the threshold for acceptable internal consistency was α ≥ 0.70 [54]. At Day 42, relationships between infant weight, formula intake, and ITQOL, ICQ and BEBQ scales were assessed using Spearman’s correlation coefficients. *P*-values less than 0.05 were considered to be statistically significant for all tests.

## Results

### Participant characteristics

**Fig. 1:**
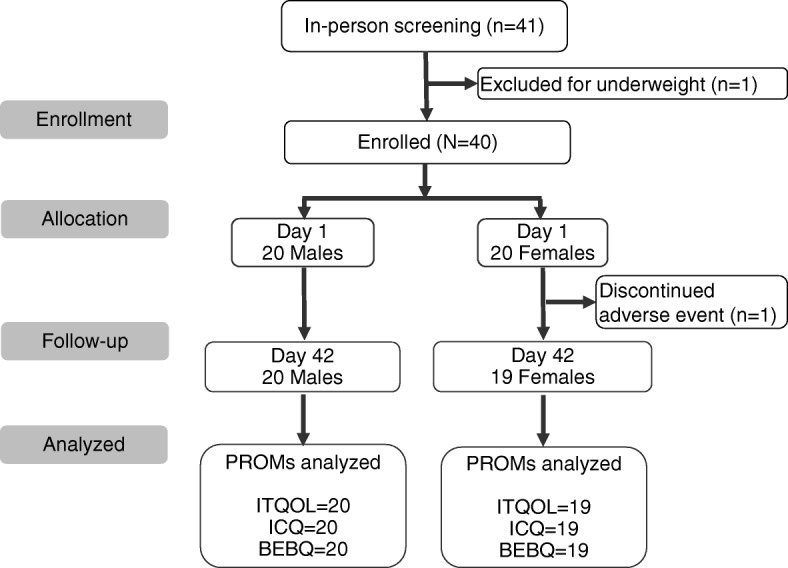
Disposition of study participants

Of the 41 infants screened for eligibility, one was excluded for being underweight and one discontinued due to an unrelated AE (gastroesophageal reflux disease). Study attrition was low (2.5%) (Fig. 1). Baseline characteristics of the enrolled infants and their parents are shown in Table 1. An equal number of male (20) and female (20) infants were enrolled, with a mean ± SD gestational age of 38.9 ± 1.2 weeks and age at enrollment of 31.6 ± 2.0 days. Mothers ranged from 18 to 38 years of age (Table 1).

**Table 1:** Infant, parent and household characteristics at time of enrollment ^a^

	Male (*n* = 20)	Female (*n* = 20)	P values^b^	Total (*N* = 40)
Infant				
Age, days	31.8 ± 2.6	31.5 ± 1.8	0.54	31.6 ± 2.0
Gestational age, weeks	39.1 ± 1.1	38.8 ± 1.3	0.43	38.9 ± 1.2
Weight, gram	4274 ± 332	4061 ± 505	0.12	4167 ± 435
Length, cm	52.6 ± 1.8	51.8 ± 1.5	0.14	52.2 ± 1.7
BMI z-score	0.97 ± 1.12	0.82 ± 0.86	0.64	0.90 ± 0.99
Head circumference, cm	36.7 ± 0.8	35.9 ± 1.0	0.02	36.2 ± 0.9
Type of delivery, % cesarean	3 (15.0)	6 (30.0)	0.45	9 (22.5)
Previously breastfed, % yes	18 (90.0)	17 (85.0)	1.00	35 (87.5)
Prior breastfeeding, days	9.9 ± 7.0	9.4 ± 4.4	0.80	9.6 ± 5.8
Mother				
Mother’s age, years	25.9 ± 5.0	26.3 ± 6.1	0.82	26.1 ± 5.5
Mother’s highest level of education			0.73	
Vocational school	7 (35.0)	10 (50.0)		17 (42.5)
High school	2 (10.0)	1 (5.0)		3 (7.5)
Any college	11(55.0)	9 (45.0)		20 (50)
Employed/self-employed, % full-time	6 (30.0)	8 (40.0)	0.74	14 (35.0)
Father				
Father’s age, years	28.9 ± 6.4	29.2 ± 6.5	0.88	29.0 ± 6.3
Father’ highest level of education^c^			1.00	
Vocational school	13 (65.0)	11 (55.0)		24 (60.0)
High school	1 (5.0)	1 (5.0)		2 (5.0)
Any college	6 (30.0)	6 (30.0)		12 (15.0)
Employed/self-employed, % full-time	17 (85.0)	18 (90.0)	1.00	35 (87.5)
Household				
Number of children (less than 18 years old) living in the household			0.72	
1 child	2 (10.0)	0 (00.0)		2 (5.0)
2 children	4 (20.0)	6 (30.0)		10 (25.0)
3 children	8 (40.0)	7 (35.0)		15 (37.5)
4 children	4 (20.0)	5 (25.0)		9 (22.5)
5 or more children	2 (10.0)	2 (10.0)		4 (10.0)
Household monthly income			0.61	
10,001–20,000 Pesos	12 (60.0)	12 (60.0)		24 (60.0)
20,001–35,000 Pesos	6 (30.0)	4 (20.0)		10 (25.0)
35,001–60,000 Pesos	2 (10.0)	4 (20.0)		6 (15.0)

^a^Data presented as mean ± standard deviation or number (%);

^b^Student’s T-test or Fisher’s exact test used for *P*-value for the comparison between males and females;

^c^Father’s education, available data, *n* = 38

### Infant growth and formula intake

At Day 42 male infants were slightly heavier, Mean (SD) (5835 g ± 0.5) than females (5348 ± 0.8). From Day 1 to Day 42, the mean (SD) increase in body weight for all infants was 1451 (369) grams. At Day 1, mean (SD) BMI z-scores were not statistically different between sexes, with 12.5% (males = 3; females = 2) of the infants classified as overweight using the WHO definition [37]. While at Day 42 BMI z-scores were within the normal range (Mean, SD, Male 1.16 ± 0.5; Female 1.20 ± 1.11). Among all infants, mean BMI z-score increased by 0.28 SD units from Day 1 to Day 42 (1.18 ± 0.84) but the incidence of overweight decreased to 7.7% (males = 3). Mean daily formula intake (ml) was similar between male and female infants (908.80 ± 224.0 versus 906.91 ± 292.5, *P* = 0.256) and intake did not differ statistically when adjusted for Day 42 body weight (0.157 ±. 041 versus 0.170 ± 0.045 ml/g, respectively; *P* = 0.905). Only 4 AEs were reported during the study; none were considered related to the infant formula.

### The utility of the PROMs

#### Feasibility: Questionnaire completion

All questionnaires were self-administered and completed at the research site. Mothers completed the questionnaires in approximately 30 min (BEBQ, 3–5 min; ITQOL, up to 20 min; ICQ, 6–8 min). Infant formula intake diaries were completed at home and were returned to the study site on Day 42. The response rate was 100% for all questionnaires and diaries and there were no missing items.

### Infant toddler quality of life questionnaire™: HRQoL

The utility of the ITQOL was also assessed by measuring the instrument’s reliability. Table 2 summarizes the ITQOL infant- and parent-concept scale scores at Days 1 and 42. Overall, the majority of infant- and parent- concept scales were reliable with Cronbach’s alpha ≥0.70 except for General Health Perceptions at Days 1 and 42 and Temperament and Mood at Day 42. Median scale scores were ≥ 80 except for Temperament and Mood at Days 1 (68.0) and 42 (72.0) and General Health Perceptions at Day 1 (77). The Physical Abilities score could be calculated in only a small number of infants (Day 1, *n* = 9; Day 42, *n* = 15) due to the high percentage of items (such as sitting-up, crawling and taking steps) reported as “not doing yet”. There was a significant improvement in median Temperament and Mood scores for all infants from Day 1 to Day 42. When comparing the change in Temperament and Mood scores from Day 1 to Day 42 between female and male infants, a significant improvement was found only in the female infants’ (but not male infants’) scores. In fact, at Day 42, six out of eight median ITQOL scale scores were higher for females compared to males, but the differences were not statistically significant (all *P* > 0.05). The largest numerical changes from Day 1 to Day 42 in infant- and parent-focused concept scores (for the total sample) were in Bodily Pain/Discomfort (− 8 points) and Parental-impact Emotional (− 7 points) scores, respectively,

**Table 2:** ITQOL data at baseline (Day 1) and Day 42^a^

	Baseline, Day 1 *N* = 40		Day 42^b^ *N* = 39	
Infant-focused Concepts^c^		Cronbach’s α		Cronbach’s α
Overall Health (1 item)		NA		NA
Male	85.0 (60.0, 92.5)		85.0 (60.0, 85.0)	
Female	85.0 (85.0, 92.5)		85.0 (60.0, 100.0)	
Total	85.0 (85.0, 92.5)		85.0 (60.0, 100.0)	
Growth and Development (10 items)		0.88		0.82
Male	91.5 (84.0, 99.0)		93.0 (84.0, 100.0)	
Female	91.5 (80.0, 99.0)		98.0 (85.0, 100.0)	
Total	91.5 (81.5, 99.0)		93.0 (85.0, 100.0)	
Bodily Pain/Discomfort (3 items)		0.83		0.79
Male	100.0 (87.5, 100.0)		87.5 (71.0, 100.0)	
Female	87.5 (83.0, 100.0)		100.0 (83.0, 100.0)	
Total	100.0 (83.0, 100.0)		92.0 (75.0, 100.0)	
Temperament and Moods (18 items)		0.72		0.65
Male	67.5 (62.0, 74.0)		72.0 (67.0, 81.0)	
Female	69.5 (62.0, 78.0)		72.0 (68.0, 83.0)^d^	
Total	68.0 (62.0, 75.5)		72.0 (67.0, 81.0)^d^	
General Health Perceptions (11 items)		0.56		0.66
Male	76.0 (73.0, 84.0)		77.0 (68.0, 86.5)	
Female	81.0 (74.0, 89.0)		82.0 (77.0, 91.0)	
Total	77.0 (73.0, 87.5)		80.0 (68.0, 91.0)	
Parent- focused Concepts				
Parental Impact-Emotional (7 items)		0.95		0.88
Male	93.0 (75.0, 100.0)		82.0 (66.0, 96.0)	
Female	93.0 (84.0, 100.0)		93.0 (79.0, 100.0)	
Total	93.0 (79.0, 100.0)		86.0 (71.0, 100.0)	
Parental Impact-Time (7 items)		0.95		0.96
Male	90.0 (73.5, 100.0)		95.0 (64.5, 100.0)	
Female	90.0 (78.5, 100.0)		100.0 (76.0, 100.0)	
Total	90.0 (76.0, 100.0)		100.0 (67.0, 100.0)	
Family Cohesion (1 item)		NA		NA
Male	85.0 (60.0, 100.0)		60.0 (60.0, 92.5)	
Female	85.0 (60.0, 92.5)		85.0 (60.0, 100.0)	
Total	85.0 (60.0, 100.0)		85.0 (60.0, 100.0)	

^a^Data presented as median (Inter-quartile range, Q1, Q3) where higher concept scores represent better HRQoL

^b^Wilcoxon Sign Rank test was used to test for differences in change from Day 1 to Day 42 by sex and total

^c^Physical abilities concept not included in the analysis due to large number of items marked as “not doing yet”; Day 1, *N* = 9 and Day 42, *N* = 15

^d^Significant at α level of < 0.01

NA, not applicable; Cronbach’s alpha cannot be calculated for overall health and family which are assessed with single items

### Infant characteristics questionnaire: Infant temperament

ICQ factor scores are shown in Table 3. There were no significant differences in ICQ factor scores between male and female infants. Cronbach’s α for all factor scales were borderline-low at Day 1 (range α = 0.55 to 0.66). Only Fussy-Difficult and Unpredictable factor scales showed acceptable reliability at Day 42 (α = 0.74 and 0.71, respectively). Median scores for all infants improved significantly from Day 1 to Day 42 for all factors except Fussy-Difficult, which had the least favorable median score (15.5) at baseline and did not improve significantly at Day 42 (P > 0.05).

**Table 3:** ICQ Factor Scores by Visit^a^

	Baseline, Day 1 *N* = 40		Day 42^b^ *N* = 39	
ICQ Factors		Cronbach’s α		Cronbach’s α
Fussy-difficult (6 item)		0.67		0.74
Male	15.5 (14.0, 20.0)		15.0 (11.0, 18.0)	
Female	15.5 (13.0, 19.0)		14.0 (11.0, 17.0)	
Total	15.5 (13.5, 19.0)		14.0 (11.0, 18.0)	
Unadaptable (4 items)		0.62		0.69
Male	10.5 (7.0, 14.0)		10.5 (7.0, 12.0)	
Female	11.5 (7.5, 15.0)		10.0 (7.0, 11.0)^c^	
Total	10.5 (7.0, 15.0)		10.0 (7.0, 12.0)^c^	
Dull (3 items)		0.65		0.29
Male	10.5 (9.0, 12.0)		9.0 (8.5, 9.5)^c^	
Female	11.5 (9.0, 13.5)		9.0 (7.0, 11.0)^c^	
Total	11.0 (9.0, 13.0)		9.0 (8.0, 10.0)^c^	
Unpredictable (3 items)		0.55		0.71
Male	8.0 (4.0, 9.0)		6.0 (4.5, 8.0)	
Female	8.0 (6.0, 10.5)		6.0 (5.0, 9.0) ^c^	
Total	8.0 (6.0, 9.0)		6.0 (5.0, 8.0)^c^	

^a^Data presented as median (inter-quartile range, Q1, Q3) where lower scores are optimal and higher scores indicate greater expression of the ICQ factor;

^b^Wilcoxon Sign Rank test was used to test for differences in change in median from Day 1 to Day 42 by sex and total;

^c^Significant at α level of < 0.05

### Baby eating behaviour questionnaire: Measure of appetite

BEBQ scores at Day 42 are displayed in Table 4. Median scores for all scales were similar in males and females (*P* > 0.5 for all). Cronbach’s α was unacceptably low for Slowness in Eating (α = 0.48) and Satiety Responsiveness (α = 0.25). Enjoyment of Food scores were negatively skewed, with mostly high scores reported for both males and females. The single-item General Appetite score was also high (4.72 ± 0.7) for all infants, while Slowness in Eating was positively skewed with a predominance of low scores (1.71 ± 0.6) indicating few of the infants were perceived as slow eaters.

**Table 4:** BEBQ Appetite Trait Scores at Day 42^a^

BEBQ Subscale^b^	Male (*N* = 20)	Female (*N* = 19)	Total (*N* = 39)	Cronbach’s α
Enjoyment of Food^c^ (4 items)				0.73
Median (IQR)^d^	4.75 (4.38, 5.08)	4.75 (4.50, 5.00)	4.75 (4.50, 5.00)	
Mean ± SD	4.61 ± 0.51	4.57 ± 0.70	4.59 ± 0.60	
Food Responsiveness (6 items)				0.77
Median (IQR)	3.58 (3.17, 4.25)	3.50 (3.00, 4.00)	3.50 (3.0, 4.17)	
Mean ± SD	3.60 ± 0.81	3.45 ± 0.82	3.53 ± 0.81	
Slowness in Eating^c^ (4 items)				0.48
Median (IQR)	1.50 (1.13, 2.13)	1.75 (1.25, 2.25)	1.75 (1.25, 2.25)	
Mean ± SD	1.65 ± 0.58	1.78 ± 0.62	1.71 ± 0.60	
Satiety Responsiveness (3 items)				0.25
Median (IQR)	2.33 (1.83, 3.50)	2.33 (2.33, 3.00)	2.33 (2.00, 3.00)	
Mean ± SD	2.48 ± 0.958	2.53 ± 0.405	2.50 ± 0.733	
General Appetite^c^ (1 item)				NA^e^
Median (IQR)	5.00 (4.50, 5.00)	5.00 (5.00, 5.00)	5.00 (5.00, 5.00)	
Mean ± SD	4.75 ± 0.44	4.68 ± 0.82	4.72 ± 0.65	

^a^Higher scores indicate stronger expression of the specific appetite trait

^b^Mann Whitney U Test was used for between sex comparisons, no significant differences found, *P* > 0.05;

^c^Scales were non-normally distributed, mean ± SD shown for comparisons with other study populations;

^d^IQR: Inter-quartile Range (Q1, Q3);

^e^NA, not applicable; Cronbach’s α cannot be calculated for single items

### Relationships between infant HRQoL, temperament and eating behaviors and formula intake and infant weight

There were no significant associations between formula intake (ml/day), change in infant weight (g) between Days 1 and 42, and BMI z-score at Day 42 with ICQ Fussy-Difficult score or ITQOL Temperament and Moods score at Day 42. There was a moderately negative, significant association between formula intake and BEBQ Satiety Responsiveness (*r* = − 0.358, *P* = 0.025), with higher formula intake associated with lower Satiety Responsiveness, a food avoidance behavior (Table 5).

**Table 5:** Spearman’s correlation between change in weight, BMI Z-scores, infant formula intake, ITQOL Temperament and Moods, ICQ Fussy-Difficult and BEBQ appetite traits at study day 42

At Study Day 42	ITQOL TM	ICQ FD	ICQ DULL	ICQ UA	ICQ UP	BEBQ EF	BEBQ FR	BEBQ SE	BEBQ SR	BEBQ GA
Change in weight from Day 1	0.064	− 0.082	0.058	− 0.122	0.117	− 0.057	0.058	− 0.275	0.198	0.124
BMI Z-score	0.268	−0.106	− 0.259	− 0.249	− 0.108	0.089	−0.022	− 0.176	−0.131	0.235
Formula Intake, ml/day	−0.223	0.107	0.212	0.148	0.106	−0.194	−0.011	0.165	−0.358^a^	0.045
Formula intake, weight adjusted, ml/g body weight	−0.298	0.089	0.182	0.178	0.029	−0.182	−0.097	0.251	−0.311	− 0.065
ITQOL TM	1	−0.488^b^	−0.377^a^	− 0.434^b^	−0.465^b^	0.192	−0.030	− 0.455^a^	−0.072	0.236
ICQ FD		1	0.128	0.594^b^	0.534^b^	−0.243	− 0.023	0.230	− 0.253	−0.003

^a^Spearman’s correlation coefficient is significant at an α level of < 0.01

^b^Spearman’s correlation coefficient is significant at an α level of < 0.05

Abbreviations: *ITQOL TM,* ITQOL temperament and moods; *ICQ FD,* ICQ fussy-difficult; *ICQ UA,* ICQ unadaptable; *ICQ UP,* ICQ unpredictable; *BEBQ EF,* BEBQ enjoyment of food; *BEBQ FR,* BEBQ food responsiveness; *BEBQ SE,* BEBQ slowness in eating; *BEBQ SR, BEBQ* satiety responsiveness, and *BEBQ GA,* BEBQ general appetite

#### Correlations between infant temperament and BEBQ scores

Difficult infant temperament measured with the ICQ was not significantly correlated with any BEBQ appetite traits. However a significant, moderate, negative correlation was found between the ITQOL Temperament and Mood concept score and Slowness in Eating (*r* = − 0.455, *P* < 0.01) (Table 6).

**Table 6:** Spearman’s correlation between ITQOL Infant and Parent/Family-focused Concepts and the BEBQ Baby Eating Behaviour appetite traits at Day 42^a^

	BEBQ Baby Eating Behaviour Appetite Traits				
	Enjoyment of Food	Food Responsiveness	Slowness in Eating	Satiety Responsiveness	General Appetite
ITQOL Infant-focused Concepts^c^					
Overall health	0.275	0.099	−0.356^b^	0.042	0.252
Growth and Development	0.348^b^	0.106	−0.407^b^	0.083	0.538^a^
Bodily Pain/Discomfort	0.291	0.048	−0.462^a^	0.143	0.262
Temperament and Moods	0.192	−0.030	−0.445^a^	− 0.072	0.236
General Health Perceptions	0.414^a^	0.173	−0.319^b^	−0.243	0.225
ITQOL Parent-focused Concepts					
Parental Impact-Emotional	0.372^b^	−0.031	−0.344^b^	− 0.104	0.336^b^
Parental Impact-Time	0.416^a^	0.164	−0.473^a^	− 0.086	0.322^b^
Family Cohesion	0.181	0.243	− 0.106	−0.102	0.219

^a^Spearman’s correlation coefficient is significant at an α level of < 0.01;

^b^ Spearman correlation coefficient is significant at an α level of < 0.05

^c^Physical abilities concept not included in the analysis due to large number of items marked as “not doing yet”

#### Correlations between BEBQ and ITQOL scores

In addition to being correlated with the ITQOL Temperament and Mood score at Day 42, Slowness in Eating also was shown to be significantly (*P* < 0.05) moderately negatively correlated with all but 1 of the remaining ITQOL concepts assessed in this study (Table 6). Additionally, Enjoyment of Food and General Appetite scores were positively correlated with Growth and Development, Parental Impact-Emotional and Parental Impact-Time scores. Enjoyment of Food was also positively associated with General Health Perceptions. Food Responsiveness and Satiety Responsiveness were not correlated with any ITQOL infant-focused or parent-focused concept scores.

## Discussion

This is the first study to examine the utility of the Tagalog translations of the ITQOL, ICQ and BEBQ to evaluate infant HRQoL, temperament and eating behavior, respectively. High response rates and lack of missing items provide preliminary evidence of the feasibility of administering these PROMs. However, the unacceptable reliability of several ICQ and BEBQ scales, along with the limited ability to calculate ITQOL Physical Abilities scores, suggests that certain scales might be problematic. This study found no differences in formula intake or most PROM scale scores in male and female infants. Infants in this study trended towards less difficult temperament and generally high (≥80) HRQoL. In addition, HRQoL was negatively associated with Slowness in Eating, and positively associated with Enjoyment of Food and General Appetite.

The ICQ was used to assess infant temperament in this study. Although some of the ICQ’s scales were found to have unacceptable internal consistency, the significant improvements in ICQ Unadaptable, Unpredictable and Dull scores suggest general improvements over the 6-week study period in mothers’ perceptions of the predictability of their infant’s behavior and the infant’s social and environmental responsiveness (i.e., smiles and happy sounds, excitement) and activity. These positive developmental changes would be expected to occur in infants 2.5 months of age [55]. The only ICQ factor that did not change significantly from Day 1 to Day 42 was the Fussy-Difficult scale, which assesses the infant’s susceptibility to distressful behaviors (i.e., fussiness, soothability and “how easily upset”) that may act as potential triggers for feeding practices associated with accelerated weight gain in infants [11, 22, 39].

The mothers in our study perceived their 2.5–month-old infants to have low levels of difficult temperament [mean ICQ Fussy-Difficult score 14.6]. To the best of our knowledge the ICQ has not been administered in any other Asian populations. However, an earlier study conducted in the US reported notably higher Fussy-Difficult factor scores (mean 24.7 to 25.8) in healthy, formula-fed infants, approximately 2.5 months of age [56]. An additional study conducted in the US [57] reported even higher ICQ Fussy-Difficult scores (32.2 to 44.5) in a population of infants described by their parents as being “very fussy” or “extremely fussy”. Thus, the ICQ Fussy-Difficult scale appears to be sensitive to differences in maternal perception of Fussy-Difficult temperament between healthy and “very or extremely fussy” American infants. Filipino mothers in the present study rated their infants’ temperament more favorably than the parents in either of the 2 studies conducted in the US. It is possible that the difference in scores reflects true differences in the fussiness of the infants included in the study samples. Although, this study administered a culturally adapted translation of the English-language version of the ICQ it is not possible to determine if the difference in Fussy-Difficult scores in the US and Filipino study populations reflects a misinterpretation of concepts, a lack of conceptual equivalence in translation or true social and cultural differences in the way mothers interpret and rate difficult infant behaviors. Furthermore, it is unknown if the wording and structure of the ICQ amplified sociocultural differences between study populations with respect to maternal ratings [44]. The ICQ’s instructions ask mothers to “circle the number that is most typical of your baby” and define “about average” as the value that reflects how the mother thinks “the typical baby would be scored.” Subsequent items are structured with a 7-point Likert response format, anchored by “very easy” and “difficult,” with “about average” in the middle. Thus, mothers are asked to rate their infants’ characteristics in the context of what they believe to be a “typical baby.” Further research is warranted to validate fussiness scores against objective data and observed behaviors (e.g., crying, time to soothe) in a Filipino sample and to fully explore cross-cultural factors that influence parents’ perceptions of infant temperament.

This study also evaluated infant temperament and other aspects of infant HRQoL using the linguistically validated Tagalog translation of the ITQOL. Western language versions of the ITQOL have been used successfully to measure HRQoL in young infants [58–61]. Yet only one study has administered the ITQOL in healthy, young, Asian infants. In an observational cohort of Chinese infants ages 42 to 90 days, Hays et al. reported similarly high scores (> 75) for all ITQOL concept scales except Temperament and Moods [62]. As observed in the present study, Hays et al. also reported an improvement in Temperament and Mood scores over a similar period of time.

In contrast, the present study failed to detect a significant improvement in the ICQ Fussy-Difficulty score. Furthermore, our data did not support the hypothesis that difficult infant temperament is associated with greater food responsiveness and lower food avoidance. Nonetheless, better ITQOL Temperament and Mood scores were associated with lower levels of Slowness in Eating. In the absence of ICQ or ITQOL reference data in Filipino infants, the discrepancies in findings for the ICQ and ITQOL temperament measures may be explained by comparing the conceptual framework for both instruments. The ITQOL was developed based on the WHO’s definition of health as “a state of complete physical, mental, and social well-being and not merely the absence of disease” [63]. It was constructed to be a generic measure of infant and toddler HRQoL for the measurement of numerous dimensions of health. Therefore, the items included in the ITQOL Temperament and Mood concept comprise a comprehensive representation of the construct of Temperament and Mood and include both positive and negative attributes, whereas the conceptual framework of the ICQ was narrower. It was primarily based on the measurement of infant difficultness, with the Fussy-Difficult factor covering mostly negative aspects of the Fussy-Difficult construct. Nevertheless, both the ITQOL Temperament and Mood and ICQ Fussy-Difficulty scales include conceptually overlapping items (i.e., fuss, respond, upset, difficult), which were shown in this study to be moderately correlated. Further studies assessing the psychometric properties of the ICQ and ITQOL may provide a better understanding of the nature of this relationship.

The findings of this study support associations between better infant HRQoL and better appetite, including greater Enjoyment of Food; higher levels of General Appetite, and lower levels of Slowness in Eating. In general, mothers gave high ratings for their infants’ HRQoL and perceived their infants as enjoying their feedings, reporting higher scores for the food approach appetite traits, Enjoyment of Food and Food Responsiveness, and lower scores for the food avoidance traits, Slowness in Eating and Satiety Responsiveness. The significant relationship between formula intake and Satiety Responsive also lends support for the finding of a generally healthy appetite. However, the unacceptable Cronbach’s α (*r* = 0.25) for Satiety Responsiveness, which may reflect the small number of items in the scale, limited our ability to accurately interpret the relationship between HRQoL, Satiety Responsiveness and formula intake. A similar weakness with the Satiety Responsiveness scale was also detected in the English version of the questionnaire [40]. Despite the limited number of studies administering the BEBQ, with the exception of the scores for Food Responsiveness, the BEBQ response pattern found in this study is consistent with those of the GEMINI Twin Birth Cohort [37] and a community-based sample of Australian infants [40]. In our study, Filipino mothers rated their infant’s Food Responsiveness higher than similarly aged infants in Australia [40] or England and Wales [37]. Food responsiveness reflects the mother’s perception of the infant’s response to food and how she responded to questions such as “My baby frequently wants more milk than I provide,” “If allowed to, my baby would take too much milk,” and “If given the chance, my baby would always be feeding.” Other studies have reported that high Food Responsiveness and lower Satiety Responsiveness is characteristic of a “heartier appetite” in infants [41] and associated with more rapid growth during infancy [64, 65].

In this present study, although the incidence of overweight infants declined from 12.5 to 7.7%, we failed to detect a relationship between weight status and HRQoL, temperament and appetite. These results are consistent with the findings from a randomized controlled trial in Filipino infants that did not detect a significant relationship between temperament and weight in infants 3 months of age measured with the Carey Early Infant Temperament Questionnaire [65]. In contrast, other studies conducted in Western and Asian infant populations report significant relationships between difficult temperament, certain appetite traits and infant behaviors (e.g., negative reactivity, soothability, self-regulation and distress), and early weight gain and childhood weight status [11, 17–20, 41, 50, 64, 66]. Differences in infant age, assessment tools and their cultural equivalence, presence of family support, cultural beliefs, and other unmeasured factors may contribute to the discrepancies between study results. In addition, the observation that the infants in our study did not experience a pattern of rapid weight gain may have contributed to our failure to detect a relationship; however, we cannot rule out other environmental, behavioral or genetic factors. Therefore, based on the data from this study, we cannot draw any definitive conclusion about the relationship between HRQoL, infant temperament and appetite traits, and infant weight status.

### Strengths and limitations

This is the first study to test the feasibility and utility of the Tagalog translation of the ITQOL, ICQ and BEBQ in a prospective cohort of Filipino infants. The critical appraisal of feasibility is an important first step towards identifying operational challenges and to support the scientific developmental work needed to examine the individual scale scores for each questionnaire, the response patterns and their interpretation. Furthermore, the preliminary data from this study may be used to inform sample size power calculations and may provide an understanding of the cultural relevance of the Tagalog translation of the PROMs. Limitations of this study included a homogenous healthy population, and an uncontrolled study design. While the study’s small sample size limited our ability to examine the relationship between potential confounding factors such as parenting style, birth order and number of siblings on infant HRQol, temperament and eating behaviors. The data were collected at one study site in Manila from formula-fed infants, which limits the generalizability of the findings to breast-fed infants and infants in other regions in the Philippines.

## Conclusion

The present study demonstrated the feasibility and potential utility of the ITQOL, ICQ and BEBQ for use in young infants in the Philippines. The questionnaires were easy to administer, completed by participants without any difficulty and successfully scored. Yet, a number of the individual PROMs scales were shown to have low internal consistency requiring further adaptations for use in different populations. Preliminary data indicated no sex differences in formula intake, infant weight, and most questionnaire scale scores. Overall, mothers perceived their infants as having positive HRQoL across a wide range of infant-focused and parent-focused concepts and a trend towards improved infant temperament over time. Furthermore, infants’ eating behaviors were generally consistent with hearty appetites. This study did not detect a relationship between infant temperament and infant weight gain or BMI at 2.5 months but did find an association between several aspects of HRQoL and eating behaviors and between Satiety Responsiveness and formula intake. There are a lack of culturally-relevant outcome measures and studies examining parental perceptions of infant behaviors, temperament and HRQoL in Asia. Preliminary data from this study support the potential utility of these questionnaires in future studies assessing cross-cultural differences in HRQoL, temperament and appetite and the influence of these factors on parent-infant interactions, feeding practices and infant weight.

## Abbreviations

AE: Adverse event
BEBQ: Baby eating behaviour questionnaire
BMI: Body mass index
HRQoL: Health-related quality of life
ICQ: Infant characteristics questionnaire
ITQOL: Infant and toddler quality of life questionnaire
PROM: Patient-reported outcome measure
WHO: World Health Organization
